# An integrated RH map of porcine chromosome 10

**DOI:** 10.1186/1471-2164-10-211

**Published:** 2009-05-08

**Authors:** Jian-Gang Ma, Hiroshi Yasue, Katie E Eyer, Hideki Hiraiwa, Takeshi Shimogiri, Stacey N Meyers, Jonathan E Beever, Lawrence B Schook, Craig W Beattie, Wan-Sheng Liu

**Affiliations:** 1 Department of Dairy and Animal Science, College of Agricultural Sciences, Pennsylvania State University, 305 Henning Building, University Park, PA, USA; 2 Key Laboratory of Biomedical Information Engineering of Ministry of Education, School of Life Science & Technology, Xi'an Jiaotong University, Xi'an, PR China; 3 Genome Research Department, National Institute of Agrobiological Sciences, Ikenodai, Tsukuba, Ibaraki 305-0901, Japan; 4 Department of Biology, College of Sciences, University of Nevada, Reno, NV, USA; 5 Faculty of Agriculture, Kagoshima University, Korimoto, Kagoshima 890-0065, Japan; 6 Department of Animal Science, University of Illinois at Urbana-Champaign, Urbana, IL, USA; 7 Department of Surgical Oncology, Room 618 820 CSB, University of Illinois COM, 840 South Wood St, Chicago, IL, USA

## Abstract

**Background:**

Whole genome radiation hybrid (WG-RH) maps serve as "scaffolds" to significantly improve the orientation of small bacterial artificial chromosome (BAC) contigs, order genes within the contigs and assist assembly of a sequence-ready map for virtually any species. Here, we report the construction of a porcine: human comparative map for pig (*Sus scrofa*) chromosome 10 (SSC10) using the IMNpRH2_12,000-rad _porcine WG-RH panel, integrated with the IMpRH_7000-rad _WG-RH, genetic and BAC fingerprinted contig (FPC) maps.

**Results:**

Map vectors from the IMNpRH2_12,000-rad _and IMpRH_7,000-rad _panels were merged to construct parallel framework (FW) maps, within which FW markers common to both panels have an identical order. This strategy reduced map discrepancies between the two panels and significantly improved map accuracy. A total of 216 markers, including 50 microsatellites (MSs), 97 genes and ESTs, and 69 BAC end sequences (BESs), were ordered within two linkage groups at two point (2 pt) LOD score of 8. One linkage group covers SSC10p with accumulated map distances of 738.2 cR_7,000 _and 1814.5 cR_12,000_, respectively. The second group covers SSC10q at map distances of 1336.9 cR_7,000 _and 3353.6 cR_12,000_, yielding an overall average map resolution of 16.4 kb/cR_12,000 _or 393.5 kb per marker on SSC10. This represents a ~2.5-fold increase in map resolution over the IMpRH_7,000-rad _panel. Based on 127 porcine markers that have homologous sequences in the human genome, a detailed comparative map between SSC10 and human (*Homo sapiens*) chromosome (HSA) 1, 9 and 10 was built.

**Conclusion:**

This initial comparative RH map of SSC10 refines the syntenic regions between SSC10 and HSA1, 9 and 10. It integrates the IMNpRH2_12,000-rad _and IMpRH_7,000-rad_, genetic and BAC FPC maps and provides a scaffold to close potential gaps between contigs prior to genome sequencing and assembly. This map is also useful in fine mapping of QTLs on SSC10.

## Background

Radiation hybrid (RH) mapping is an important tool in the construction of high-resolution physical maps, which are key to efficient sequencing and successful genome sequence assembly [1-5] and the construction of comparative maps between species [6-8]. A major advantage of RH mapping is that polymorphic and nonpolymorphic markers, including sequence tagged-sites (STSs), microsatellites (MSs), genes, expressed sequence tags (ESTs), bacterial artificial chromosome (BAC) end sequences (BESs) and single nucleotide polymorphisms (SNPs) can be ordered at high resolution. Although several RH panels are currently available for swine [1,9-14], to date, the IMNpRH2_12,000-rad _panel [1] offers the highest mapping resolution and has been successfully used to order a variety of markers on swine chromosome (SSC) 2p, 9p, 12 [15,16], and over several chromosome regions, SSC6q1.2 [17], SSC7q11-14 [18], and SSC15q25 [1]. Current RH maps of SSC10 are built on the IMpRH_7,000-rad _panel [8,19-22] where map resolution and marker density are unfortunately not optimal for either fine-mapping QTLs or genome sequence assembly. Map discrepancies between various RH maps or between RH and the corresponding genetic map(s) also exist [7,22-24]. Therefore, an integrated, high-resolution RH and comparative map for SSC10 should help in resolving discrepancies.

SSC10 is a small metacentric chromosome, comprising ~3.1% (85/2700 Mb) of the pig haploid genome. Sequencing of SSC10 by the International Swine Genome Sequencing Consortium (SGSC) and Pig Genome Sequence Project  is based on the BAC fingerprinted contig (FPC) map  and the BES RH map [25,26], which was built on the IMpRH_7,000-rad _panel. An initial draft assembly of SSC10 sequence has recently been released in Pre-Ensembl , but only contains ~30 Mb, roughly one-third of the entire chromosome. Here we report the generation of an initial high-resolution RH map of SSC10 that integrates the IMpRH_7,000-rad_, IMNpRH2_12,000-rad _RH, porcine genetic and BAC FPC maps, and allowed us to construct a comparative map of SSC10 and HSA1, 9 and 10.

## Results and Discussion

### SSC10 RH_12,000 _and RH_7,000 _maps

**Figure 1:**
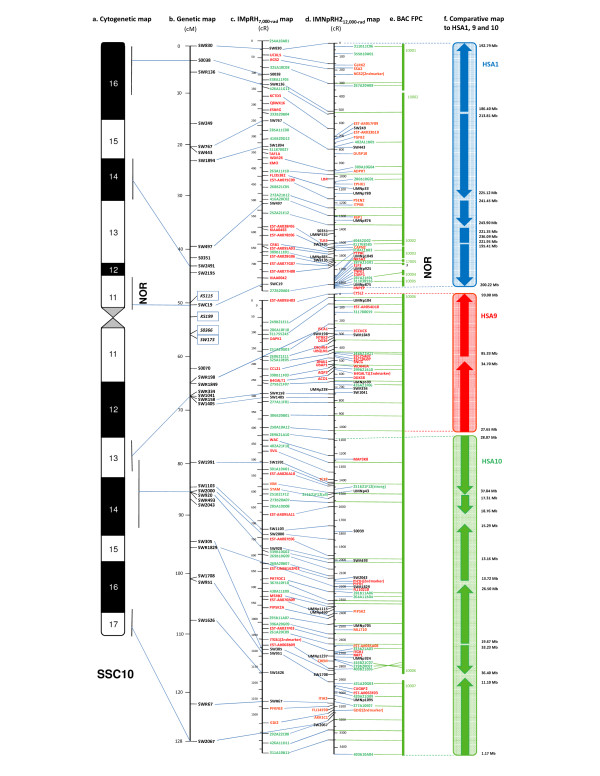
**High-resolution RH comprehensive and comparative maps of SSC10**. (a) Cytogenetic map of porcine chromosome (SSC) 10. The nucleolus organizer region is indicated as NOR; (b) Genetic map of SSC10. Those MS markers that are mapped in the current 7,000- and 12,000-rad FW maps and non-FW maps are listed in the genetic map. The four singletons that were not in FW maps are marked in italics in a box; (c) The 7000-rad IMpRH framework (FW) map; (d) The 12,000-rad IMNpRH2 FW map. MSs highlighted in black, genes/ESTs in red, and BESs in magenta. Markers listed between the 7,000- and 12,000-rad FW maps are framework markers; those on the right of the IMNpRH2 FW map are non-framework markers; (e) BAC fingerprinted contig (FPC). Contig number, e.g. 10001, corresponds to the FPC maps at ; Question mark (?) indicates that the BES 287A21G01 is lack of information in the FPC maps. (f) Comparative to human chromosome (HSA) 1, 9 and 10. Synteny blocks between pig and human, as well as the orientation of the human sequences in reference to the SSC10 RH map, are indicated by arrows, with HSA1 in blue, HSA9 in red and HSA10 in green. Sequence position of each synteny block in the human genome sequences (Build 36.3) is listed (in Mb) on the right side of the chromosomes.

Two sets of mapping vectors from the IMNpRH2_12,000-rad _and the IMpRH_7,000-rad _panels were used to construct the RH FW map of SSC10. The vectors were merged into one data-set and analyzed using a maximum multipoint likelihood linkage strategy with CarthaGene [27] at a 2 pt LOD score of 8. Two linkage groups were initially identified for SSC10 (Table 1, 2). One linkage group was assigned to the short arm (p), the other group to the long arm (q) (Fig. 1c–d) based on known MSs and ESTs/genes from previous genetic and RH maps [6,7,14,21,25,28]. A FW map for each linkage group was built simultaneously on both RH panels at a likelihood ratio of 1000:1. The orientation of each linkage group was based on markers previously assigned to the porcine cytogenetic (Fig. 1a) and genetic maps (Fig 1b). A total of 107 FW markers common to the IMpRH_7,000-rad _(Fig. 1c) and IMNpRH2_12,000-rad _(Fig. 1d) FW maps were ordered on SSC10 (Table 1, 2). The accumulated map distance of SSC10 was 2075.1 cR_7,000 _on the IMpRH_7,000-rad _and 5168.1 cR_12,000 _on the IMNpRH2_12,000-rad _FW maps. This represents a 2.5 fold increase in map resolution over the IMpRH_7,000-rad _panel (Table 1), which is consistent with our previous observations on SSC2p, 9p and 12, [15,16], and within the 2.2–3.0 fold range reported on SSC6q1.2 [17], SSC7q11-14 [18], and SSC15q25 [1]. If we assume the DNA content of SSC10 is ~85 Mb [25], then the kb/cR ratio is ~16.4 for the IMNpRH2_12,000-rad _FW map, and 41 kb/cR in the IMpRH_7,000-rad _FW map, close to the genome average of 15 kb/cR_12,000 _reported for the IMNpRH2 panel [1,15], and slightly better than the 47.5 kb/cR_7,000 _genome average reported for the IMpRH_7,000-rad _panel [7]. In the second generation pig EST RH_7,000-rad _map [7], SSC10 had the lowest resolution (highest kb/cR ratio) of the entire genome with a ratio of 71.4 kb/cR_7,000_, based on an earlier estimate of ~103 Mb for SSC10 [7,28]. As chromosome size estimates (103 vs. 85 Mb; ~17% reduction) alone cannot account for the reported lower resolution in these initial reports, a lower marker density and difficulty in mapping within the nucleolus organizer region (NOR) must be considered (see discussion below).

**Table 1:** Comparison between the 12,000- and 7,000-rad RH framework (FW) maps

Linkage group	No. of marker*	**12,000-rad FW map**			**7,000-rad FW map**		Fold change**
				
		FW marker	Non-FW marker	Map distance (cR)	FW marker	Map distance (cR)	(cR_12,000_/cR_7,000_)
SSC10p	87	31	39	1814.5	41	738.2	2.5
SSC10q	129	76	49	3353.6	59	1336.9	2.5
Total	216	107	88	5168.1	100	2075.1	2.5

* Number of marker was the sum of FW and non-FW markers in both RH_12,000 _and RH_7,000 _maps (Fig. 1c & 1d).

** Fold change was calculated by dividing the map distance in the 12,000-rad FW map by the map distance in the 7,000-rad FW map.

**Table 2:** A list of markers mapped on SSC10 RH map

Marker type	RH panel	Linkage group	MS	BES	Gene/EST	Total*
FW	1,2000-rad	SSC10p	8	8	15	31
		SSC10q	16	26	34	76
	7,000-rad	SSC10p	7	15	19	41
		SSC10q	10	28	21	59
Non-FW		SSC10p	8	12	19	39
		SSC10q	13	15	21	49

*Some of the markers in each linkage group are not common markers to both RH panels, leading to the total number of markers in this table being greater than 216.

We used the CarthaGene software [27] to integrate 88 additional, non-FW markers (Table 1, 2) into the IMNpRH2_12,000-rad _FW map. They are listed to the right of the FW map in Fig. 1d (Table 1). If counting the FW and non-FW markers together, we achieved an average density of 393.5 kb per marker (85 Mb/216 STS) on SSC10, a significant increase in marker density over the ~1.1 Mb/marker reported for the most current SSC10 RH_7,000-rad _EST and BES maps [7,25].

When marker density on SSC10p and SSC10q was analyzed separately, there was a significant difference in map resolution between the two arms. The standard porcine karyotype, places the SSC10 arm ratio (q/p) at 1.2 [30] with the DNA content of SSC10p estimated at 38.7 Mb (85 × 45.5%), and SSC10q at 46.3 Mb. The kb/cR ratio of SSC10p and SSC10q would be 21.3 and 13.8 in the IMNpRH2_12,000-rad_, and 52.4 and 34.6 in the IMpRH_7,000-rad _panels, respectively. This suggests that map resolution on SSC10p was lower than that of the genome averages in both panels, whereas SSC10q was higher than the genome average [1,14-16]. There are two possible explanations for this finding. One is marker density, which is lower in SSC10p (Table 1). RH FW map length for a given region is influenced by marker density; the higher the marker density, the longer the map length (cR); thus, the higher the map resolution. A second is the localization of the NORs, or the ribosomal RNA genes (RNR, or rDNA) on SSC10p near the centromere [31-34]. SSC10 is one of two chromosomes (the other one is SSC8) in the porcine genome that harbors ribosomal RNA genes (RNR, or rDNA) in NORs located in the proximal region of SSC10p near the centromere. Silver-staining (Ag-NOR) indicates that the porcine RNR genes on SSC10 are constitutively active in all pig breeds, while their activity on SSC8 varies among different pig breeds [31]. The ratio of FW vs. non-FW markers was 1:4 (3 FW/12 non-FW) in the NOR region (Fig. 1d), significantly biased from the average 1:0.85 on SSC10 suggesting that building an accurate map for this region will be difficult. The swine BAC FPC map is also not contiguous over the same region . In the human genome, there are between 150 and 300 copies of ribosomal RNA gene per haploid genome, that map to the short arms of acrocentric chromosomes 13, 14, 15, 21, and 22 [35]. Unfortunately, the copy number of the porcine RNR gene on SSC10 is currently unknown, which limits our ability to specifically define the extent of the NOR.

It is also worth noting that a pair of markers for five genes (RGS2, B4GALT1, PHYH, ITGB1, and GDI2) was typed separately on the IMNpRH2_12,000-rad _panel. As shown in Fig. 1c and 1d, all five-paired markers mapped next to each other within a small interval. Since the paired markers were designed from different laboratories (in Japan and USA) and typed anonymously, they provide an additional indication of the resolution and accuracy of the IMNpRH2_12,000-rad _SSC10 map [15].

### Integration of SSC10 RH and genetic maps

Thirty-six of 54 (67%) MSs ordered on the IMNpRH2 panel were mapped on the latest SSC10 genetic map  (Fig. 1b), of which 23 are FW markers, 9 are non-FW markers, and the remaining 4 are singletons (a linkage group with only one marker) (Fig. 1b–d). The order of all FW and non-FW MS, except for SWR1829 on the SSC10 RH map, is in agreement with the order on the genetic map (Fig. 1b–d). In addition, the five bins (≤ 5 cM), each with two to five MSs, on the genetic map (USDA-MARC v. 2.) of SSC10 (Fig. 1b)  were all ordered on the IMNpRH2_12,000-rad _map (Fig. 1c and 1d), significantly increasing the resolution on the genetic map.

However, two small regions of SSC10 did not completely align with the genetic map. The first is the NOR-centromere region. The four singleton MSs (KS115, KS199, S0366, and SW173) (Fig. 1b) were all located in this region. The second region is close to SSC10qter between SW2043 and SW1626, where there is a gap between FPC contigs 10006 and 10007. We determined MS SWR1829 and SW305 were flipped when compared to the genetic map (Fig. 1b–d). In previous studies, SWR1829 and SW2043 were reversed [22,24], while the order of SW951 and SW305 was reversed in the IMpRH_7,000-rad _maps for SSC10 reported by Aldenhoven et al. [24] and Rink et al. [7], indicating the difficulty in assigning marker order within this region.

### Integration of SSC10 RH and BAC FPC maps

When the SSC10 RH map was integrated with the corresponding BAC FPC maps based on common BES (Fig. 1c–e), a total of 7 BAC contigs (10001–10007) were identified. Contigs 10001–10005 cover SSC10p, while contigs 10006–10007 cover the entire SSC10q (Fig. 1e) with the order of BESs in the current RH map identical to the FPC map for the entire chromosome  except for the two small regions also encountered in the genetic map. We estimate the 4 singleton MSs (KS115, KS199, S0366, and SW173) in the genetic map, the NOR and centromere region of SSC10 cover ~5.5 cM (from 50 cM to 55.5 cM) (Fig. 1b) with the NOR between 1550 cR_12,000-rad _and 1850 cR_12,000-rad _(Fig. 1c) where several small BAC FPCs including contig 10003, 10004 and 10005 are assigned. There were two "free" BES markers found between contigs 10003 and 10004 in the RH map (Fig. 1e). One was 309B11E01, which mapped to the middle of the large FPC contig 17005 . The other was 287A21G01, for which no information is available in the BAC FPC database. It is worth noting that since neither RH mapping nor BAC fingerprinting were able to accurately map the NOR region, more attention should be paid to marker assignment and sequencing in this "problematic" region.

The order of three non-FW BESs (416B21C07, 278B20E07, 403B21E06) at the end of BAC contig 10006 was also inverted (Fig. 1e). Four FW MSs, SW305, SW1708, SW951 and SW1626 are located in this region where discrepancies between the genetic and RH maps were previously reported [7,23,24]. CarthaGene identified two likely FW map orders for this region, with only a slight statistical difference in Log10-likelihood (-409 vs. -410). One order was CREM-ESTAR068B09-SW305-SW1708-SW951-SW1626. The alternative order was SW951-SW1708-SW305-ESTAR068B09-CREM-SW1626. The final RH map (Fig. 1c and 1d) was built according to the first order based on the assumption that marker order in the genetic map (Fig. 1b) was correct. However, adoption of the alternative order would not invert the three non-FW BESs in contig 10006. At present, we are unable to resolve whether the genetic or the FPC map is correct without additional markers.

### Comparative map between SSC10 and HSA 1, and 9 and 10

**Figure 2:**
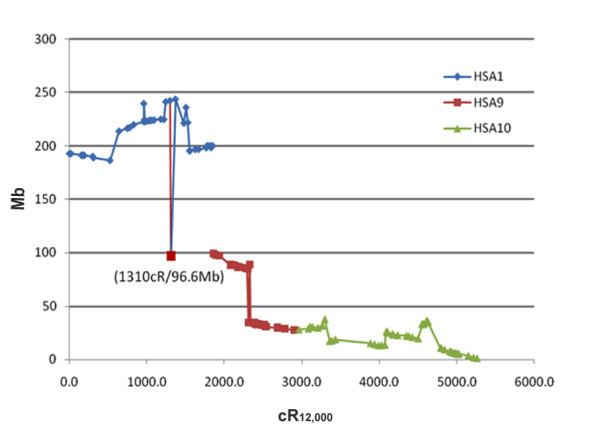
**Comparison of gene order between SSC10 and HSA1, 9, and 10**. A total of 127 genes/ESTs and BESs were compared based on their positions on SSC10 and HSA1, 9 and 10. The sequence positions (Mb) of genes on human chromosomes (Build 36.3) are compared with corresponding genes on the SSC10 RH_12,000-rad _map (Fig. 1). Map distances (cR) for SSC10 are the accumulated sum of the linkage groups from Fig. 1. Each diamond represents the Cartesian coordinates for a sequence on HSA1, squares for HSA9, and triangles HSA10. Any significant change in coordinates between adjacent genes indicates a rearrangement. The map position of FBP1 at coordinate 1310.0 CR/96.6 Mb is indicated and discussed in the text.

All gene, EST, and BES sequences were BLAT  and BLAST  searched against the human genome sequence (Build 36.3) to identify regions of synteny between the human and porcine genomes (see Additional file 1). The coordinates of 127 markers based on their positions in the current SSC10 RH map and their sequence locations in HSA1, 9 and 10 are shown in Fig. 2. The pig-human comparative map (Fig. 1) built in this work, not only supports the previous findings by bidirectional fluorescence *in situ *hybridization (Zoo-FISH) that SSC10 is in synteny with regions of HSA1, 9 and 10 [36,37], but also refines the conserved synteny blocks between the two species. Five synteny blocks are conserved between SSC10p and the distal region of HSA1q (1q31-43) from 186.4 to 243.9 Mb (Fig. 1a, f, Fig. 2). However, SSC10q shares regions of synteny with two other human chromosomes, HSA9 and HSA10. Two blocks of conserved synteny were identified between the proximal region of SSC10q (q11-12) and HSA9p13.1-21.2 (27.7 to 34.7 Mb) and HSA9q21.32-22.32 (85.3 to 97.89 Mb), while six blocks were identified between the distal region of SSC10q (q13-17.2) and the entire HSA10p (10p11-15) (1.0 to 37.8 Mb) (Fig. 1f, Fig. 2), reflecting the macro-rearrangements that occurred during the evolution of the two species. In addition, several micro-rearrangements such as those observed on SSC12, SSC2p and 9p [15,16] were also identified. For example, the FBP1 (fructose-1,6-bisphosphatase 1) gene located on HSA10q (see Additional file 1) did not map to the conserved region between SSC10 and HSA10, but to the region where SSC10 and HSA1 are conserved at 1310.0 cR/96.57 Mb (Fig. 2). It is reasonable to assume that the map position of FBP1 in SSC10 is an indication (or a relic) of an inversion in the region (1310–1860 cR_12,000_) between FBP1 and CTSL2 (cathepsin L2) (see Fig. 1d) during the evolution of SSC10 which may include the entire centromere. Two BESs, 309A10G04 at 960.0 cR/239.74 Mb and 404B2D02 at 1505.0 cR/236.09 Mb, were also not aligned with their synteny blocks. Further characterization of regions of conserved synteny as well as micro-rearrangements between pig and human should improve our understanding of mammalian genome evolution and assist in swine genome sequencing and assembly.

Gene density across a genome of any mammalian species is not uniform with some human and swine chromosomes described as "gene-rich", and others as "gene-poor" [1,2]. The first- and second-generation porcine EST RH maps [6,7] suggested that SSC10 had less than the expected number of genes based on DNA content [29]. However, none of the corresponding human chromosomes/regions (HSA1, 9, and 10) are listed as "gene-poor" in the human genome [38]. This inconsistency in gene density within homologous regions of the pig and human genome may be explained by two factors. The size of SSC10 may have been over-estimated as the initial report of 103 Mb for SSC10 [29] used in subsequent reports [6,7] has been revised to ~85 Mb [26]. Alternatively, the NOR region contains a large number of tandem repetitive DNA sequences and harbors multiple copies of the RNR genes that is likely to confound the estimate of gene density.

## Conclusion

We merged mapping vectors from the IMNpRH2_12,000-rad _and the IMpRH_7,000-rad _panels to construct the highest resolution FW RH and comparative map for SSC10, to date, containing a total of 216 markers. Average map resolution was 16.4 kb/cR_12,000 _or 393.5 kb per marker on SSC10. Although the proximal region of SSC10p (NOR-centromere region) remains a difficult target for physical as well as linkage mapping, the current map provides a platform to integrate additional STS from the wealth of data submitted to the IMNpRH2_12,000-rad _and IMpRH_7,000-rad_, genetic and BAC FPC maps. This should improve our ability to identify breakpoints and micro-rearrangements and close potential gaps between BAC contigs prior to sequencing and assembly, as well as provide a useful starting point for higher resolution mapping of QTLs including those for meat quality, growth, feed intake and reproductive traits [8,39-41] on this chromosome.

## Methods

### Genes/ESTs, MSs and BAC end markers

Primer pair sequences were acquired from normalized, essentially full-length cDNA libraries [42] or from the literature. EST markers were derived from a panel of normalized porcine cDNA libraries as described earlier [7]. BAC end primers were used essentially as reported [25]. MS primer sequences were obtained from the literature and public databases [14,28,43]. All primers were optimized by determining the highest annealing temperature at which successful amplification of porcine genomic DNA took place. Primers were then tested with both porcine and Chinese hamster genomic DNA at species-specific temperatures [15].

As discussed by Liu et al. [15], we avoided designing redundant primer pairs for a given gene. However, we identified five genes with two pairs of primers because mapping primers were designed from either the gene cDNA sequence (synthesized in Japan), or from the corresponding EST (synthesized in the USA), that were annotated only recently. The two pairs of primers were treated as two individual markers and typed separately on the IMNpRH2_12,000-rad _panel, where they served as internal controls to evaluate mapping accuracy and resolution. Additional file 1 provides detailed information regarding all markers mapped in this work.

### RH typing, data merging, and map construction

Two sets of mapping vectors from the IMNpRH2_12,000-rad _and the IMpRH_7,000-rad _panels were used to construct the RH FW map of SSC10 RH as previously described [15,16]. The first set was generated in this work on the IMNpRH2_12,000-rad _panel (Tables 1 and 2, Additional file 3). The second set was previously typed on the IMpRH_7,000-rad _panel (see Additional file 2) [7,25]. The two data sets were merged using Carthagene software [27] and merged data analyzed using a maximum multipoint likelihood linkage strategy. Each marker was assigned into linkage groups on the IMNpRH2_12,000-rad _SSC10 map at a 2 pt LOD score of 8 and a threshold distance ≤ 100 cR between markers. Common markers refer to the 13 MSs, 61 ESTs/genes and 43 BESs assigned on both panels. A framework (FW) map was constructed for each linkage group with a likelihood difference threshold of 1000:1. Additional (non-FW) markers were then mapped on the IMNpRH2_12,000-rad _panel with CarthaGene [27]. Final maps were drawn using MapCreator .

### Comparative mapping

All sequences of the porcine ESTs and BESs were BLAT  or BLAST  searched against human genome sequence (Build 36.3). Once a sequence match (95% and greater similarity of length 40 bp or more) [15] was identified, the start position of the sequence in the human genome was collected (see Additional file 1). Chromosome locations and start positions of their orthologs in the human genome were also established for all genes analyzed (Fig. 1 and 2) using the NCBI human Map Viewer (Build 36.3) . Cartesian coordinates of 127 genes/ESTs and BESs based on their map position in HSA 1, 9, and 10 in SSC10 were also developed (Fig. 2). Map distance (cR) for SSC10 in Fig. 2 were the accumulated sum of the linkage groups from Fig. 1c and 1d.

## Authors' contributions

**JGM **participated in data analysis, developed the SSC10 7000-rad and 12,000-rad RH maps, and the pig-human comparative map; the main author for drafting and revising the manuscript. **HY **participated in porcine gene isolation, primers design, RH typing on the 7000-rad panel, and data analysis. **KE **carried out RH typing on both 12,000-rad and 7000-rad panels and the consensus mapping (vector); participated in porcine EST sequencing, EST primers design, and data collection and analysis. **HH **participated in porcine gene isolation, primers design, RH typing on the 7000-rad panel, and data analysis. **TS **participated in porcine gene isolation, primers design, data collection and analysis. **SNM **participated in porcine BAC end sequencing, BES primers design, RH typing on the 7000-rad panel, and data analysis. **JEB **participated in porcine BAC end sequencing, BES primers design, RH typing on the 7000-rad panel, and data analysis. **LBS **participated in porcine BAC end sequencing, BES primers design, RH typing on the 7000-rad panel, and data analysis. **CWB **participated in the research design, coordination, porcine EST sequencing, EST primers design and mapping, and helped to revise this manuscript. **WSL**, principal author, coordinated the research project, carried out consensus mapping (vector), CarthaGene analysis, and map construction; involved in drafting the manuscript and revising it critically for important intellectual content; and edited the final version for publication.

## Supplementary Material

Additional file 1 **SSC10 marker information**. This file provides detailed information regarding all markers mapped in this work. Click here for file

Additional file 2 **IMpRH7,000-rad mapping vectors for the markers on SSC10**. This file provides mapping vector and retention frequency of all SSC10 markers in the IMpRH panel. Click here for file

Additional file 3 **IMNpRH2-12,000-rad mapping vectors for the markers on SSC10**. This file provides mapping vector and retention frequency of all SSC10 markers in the IMNpRH2 panel. Click here for file
