# Supplementary material for: An integrated RH map of porcine chromosome 10
Source: BMC Genomics. 2009 May 8;10:211. doi: 10.1186/1471-2164-10-211 (PMC2689272; doi:10.1186/1471-2164-10-211)
Supplement: Additional file 3 — IMNpRH2-12,000-rad mapping vectors for the markers on SSC10. This file provides mapping vector and retention frequency of all SSC10 markers in the IMNpRH2 panel. [file 1471-2164-10-211-S3.doc]

"Supplementary Table 2. IMpRH7,000-rad mapping vectors for the markers on SSC10*"

Marker Vector "Number of ""2""" "Number of ""1""" Retention Frequency**

311755245 001110001110001100011100011111021000100000100010000002000001011010011011101111111110011110 2 42 0.47

248A21H11 000101001110022000100100011001010101100010000000000000000001011110011111101110010110011110 2 36 0.40

249B21E11 001110010120000001011100001001111000100001100000100000200101010010011211121111111111011110 4 41 0.46

250A10A12 000001001110111011110100011210001011100000000011000000000110111100110101101210010011011110 2 41 0.46

251A10G03 000111001110011100111101011111001100200010201000000000001000011110011111101110011110011110 2 45 0.50

252A21E12 000010001101101011011100011000001000101010000010000002000001011100100111111110111010011111 1 40 0.44

254A10A01 001100100100001010010100011000000100000110100110000010000000011100100000111100010011011100 0 31 0.34

260B21E11 000111001110011100100101211011000100100010200000000000000001011100010111101110011110011110 2 39 0.43

261A20C09 000000022100000010010010011001021011000110100110111220000100111210001000111110111110111111 6 40 0.44

263A11F10 001110001101111001110110011001011000101010000010000000000001010010101010101110010012010111 1 39 0.43

264A11B04 000001000110001011010100011000101101000100101111100020000100111210011100111110111111111111 2 46 0.51

267A20H08 001100101100021000010100011001021111010001000010100000000000010010100000110100010212011100 4 30 0.33

268A20B07 000001000110011011100100011000101101000110101111110020000101010010011101111110111111111111 1 49 0.54

268B21C05 001110001101111001110110011000011000101110000010000000010101010110101111121110011010011111 1 46 0.51

269B10G09 000001000110011011100110011000101001100100001010110000001101010000010111111110111111211111 1 45 0.50

272A21B12 000010001101101011010110011000011000101010000010000201010001010010101111201110111010011111 2 41 0.46

272B20A04 000010001100011110110100011101111000100111000110001101000000001100110001101110111011011101 0 44 0.49

273B20A07 001101001111111010100000011000101001100000000110100000001102010000110011101110011011011111 1 41 0.46

275B21F07 000101001110111001110100011001010101100011011000000000001101011110011101101110010010011100 0 43 0.48

277A10E07 000000000100010010110110021211011000010111200020100012002100011000100000110110011100211101 7 33 0.37

277A11F01 000011001110100001100100012001001101102022000000000000001100012100100000101210010010012110 7 28 0.31

278B20E07 000000001100020120010110111001011011101111100110111010000100011220001000111110011110011111 4 44 0.49

281B11A06 000001000110011011100100011000101001100110101111110000000101110010011100111110111111111111 0 49 0.54

285A11C08 001110101101101001010011011200001010101101001010000000000100010000000100101110010212011110 3 36 0.40

286A10F10 001110002110000100011100001011121000100000100000000000000001011010011011101111111110011110 2 38 0.42

286B10G01 001110001101111001110110011001011000101010000020000000020001010010101011101111010010011111 2 41 0.46

287A21E01 000010011100001010000020011101111000100011000110101101000000011100110001101110111011011101 1 41 0.46

289B21A10 000001001110111001101100011011001011100101000111100000000110111000110101101100010011011110 0 44 0.49

292A22C08 000000000100010010110100011011001000011111200020001010001100011000100000100110110100011211 3 33 0.37

295B11A07 000001001100000001010010012000101111010100101110110000010102111210011100111110111110111111 3 45 0.50

299B21B10 000101001111121011100100011001010101100011000000000000000101011110011102201110010210011100 4 38 0.42

301A10H01 001101001110011010100100001010001011100001000110010000000100010000110002101110010011011110 1 36 0.40

306B20B01 000111001112111001110100011011121001101010000002100000001110111110110101101100010010011110 3 45 0.50

309A10G04 002110001101111001110110101010011010101000000010000000000001010000100010101110010010011111 1 37 0.41

309B11E01 000010001100001010010100011000001000100000000110000101000100011000110001101110111011011101 0 34 0.38

310A11B03 020010001100001000010100011100001000100100000010000001010100011100110101101110111011011101 1 36 0.40

311768585 000010001100001100011100012100001000100100000010000000000100011100100111111110111011011101 1 36 0.40

311780659 001110001110001100011100011111011100100000100010000000000001011010011011121111111110011110 1 44 0.49

311838556 000010001100001110000000012110111010100111000100001022000000001000110001101110111011011100 3 35 0.39

311870027 000110101100000001100010011010001010101000000000000000000000010000000010101110010010020111 1 26 0.29

311A10B11 000210201000101111001100011011001000010111100000011010001100011100100021101110110101011011 3 41 0.46

311B11C06 001100101100011010010100011000000100000010100110000000000000010000100020111100010212011100 3 28 0.31

325A10C08 001100101100011000110100011000001011010010000010000000000000010010100000110100010210011100 1 29 0.32

325A10E05 000101001110011001100101011011000101100010000000000000000001011100010111101110010110012210 2 36 0.40

333B21A03 000000001100002120010110011001001010001111100110111010000101111010001000111110111110011111 2 44 0.49

338A11F05 001110101100001000010100011001000101010001000010100000001000010010100000110100010211011101 1 32 0.36

333B20B04 001110101101101101010101011001120021101001000010000001001000010000100000101110010212001101 4 36 0.40

339B10G02 000001000110011011100100011000001001100101001020100000001102010000010112111210011111011111 4 38 0.42

367B10F10 000001002100000011010100011000101101000100101111110000000100111110011100112110111110111111 2 44 0.49

396A20G09 000001001100000000010000011000002011010110101110110000000100111010001000111110111110111111 1 39 0.43

398B11F03 000101001110011000100100011001010101100011000000000000000101010110011110201110010010011100 1 35 0.39

402A11B05 001110121101101101010111011001101010101101001010000001001000011000100000101110010012011110 2 42 0.47

402A21F10 000001001111111010102100000001011011100101000111100000000100111000110001101100010011011110 1 40 0.44

403B10A04 000010001000000111001100011012001000010111100000011010001100111000010001101110110101011011 1 38 0.42

403B21E06 000000001100000110010110011001011010001111100110111010000100011210001000112110011110011111 2 42 0.47

404B2D02 000010001100001011010100011000001000101010000010000002002001011100100011111110111011011111 2 37 0.41

416A20C02 000010000101101011010110011000011000101010000010000001000001011110101111011110111010011111 0 42 0.47

416B20D12 001110101100001001010010111200002010101010000010000000000100010000000010101110010112011110 3 32 0.36

416B21C07 000000002100000110010110111001111011100101100110111010000100011010001000111110011110011111 1 44 0.49

420A21D09 000000000101100011010110001011001000010110000110110110001100011000000000110110010100011101 0 35 0.39

426A11D11 000010001110001010000100011211001000010110100010001010001100011000100000101110110100211011 2 35 0.39

426A11G11 000110101000000000010100011002000101010000000010100000001000011000100000110110010010001101 1 26 0.29

426A21F06 000111001110111001100100011001000101100011000000100000001100011100110101101110010010011100 0 39 0.43

430A11E09 000001000100000001010000010000000101000100102110110000010100112000011100110110111110011111 2 34 0.38

431A20G03 000000000100000011010110000001221000000101000110111210000100011000000000111110011100011111 3 32 0.36

B4GALT1 000101001110111001110100011001010101100011000000002000000101011110011101101110010010011100 1 40 0.44

B4GALT1(secondmarker) 000101001110111001110100011001010101100011000000000000000101011100010000001110010010011100 0 35 0.39

FLJ35382 000210001101111001110110010001012000101010000010000000000100010010101011101111010010010111 2 37 0.41

CAPN2 000010001100002000010200010100001000100000000000000000000100011000000111111110111010012101 3 26 0.29

CCL21 000101001110211000110100011001010101100010010200000000000001011110011110001110010110011111 2 39 0.43

CRB1 000010001100001010010100011100001000100101000100000001000100011100210001101110111011011101 1 35 0.39

CUGBP2 000000000100001001011110000001001011100101000100110010001100011010001000111110011100011111 0 37 0.41

DAPK1 000110001110001100021100011111001100100000100020000000000001011010011010001110011110011110 2 36 0.40

DUSP10 000110101100011001110010111010001010101000000010000000000000010000000010101110010210011110 1 32 0.36

ELF3 000010001100011120100100012110211010100111000100001100000000001000110001101110111011011101 3 38 0.42

EPHX1 000110201101221001110110012001011000101010000000000000000000010010101011201111000010011111 5 34 0.38

ESRRG 001110101101101101010101011001100001002001000010000001001000011010100000101110010010001101 1 36 0.40

EST-AR026A10 001101021110011010111100011010001011100001000100010000000100010000110001101110011011011110 1 40 0.44

EST-AR028G06 000010001100021010011000011000001000100000000100000100000100011100110001101110111011011101 1 33 0.37

EST-AR033B10 000110101101110101010101011000101011101001001010000000011100010000100000101100010010011110 0 38 0.42

EST-AR037F03 000001001100001010012010011001101011000110101110110000000100111010001000111110111110111111 1 44 0.49

EST-AR038F06 000010001100001010011100010100101000100100000000000000000100011100110101111110111011011101 0 36 0.40

EST-AR054D10 000110001110001100011100011011011000100000100000000000000001011010011011101111111110011110 0 40 0.44

EST-AR057F09 000110101101110101000101010000001011101001000000000000011100010000100000000100012010010110 1 30 0.33

EST-AR063F03 000000000100000001011110010001001011100101000100111010001100011020001000111110011100011011 1 36 0.40

EST-AR068B09 000000000100000100010110010001010010001111100110111010010100011012001000110010011110011111 1 38 0.42

EST-AR075C09 000110001101111001110110011001001000101010000010000000000100010000101011101110011010011111 0 39 0.43

EST-AR076B09 000001000100000001011010010000000101010100101110110000010100111010011100110010111110111111 0 40 0.44

EST-AR077G07 000022000200000010010000011001001000100001000100001100000100011000110001101110110011010100 3 27 0.30

EST-AR077H08 000010001100001100011000010001001000100001000100001101000100011000110001101110111011011101 0 35 0.39

EST-AR078E06 000010001100000000010100010100001000100000000000000000000100010000000201101110111010011202 3 22 0.24

EST-AR085A08 000000000100000100010010010001000010001111100010110010000100110000000000010000110110010011 0 27 0.30

EST-AR087E06 000001000110001001100100010000001001000101000000000000001100010000010110110010011111011111 0 31 0.34

EST-AR095A03 000010001100001010011100011100001000100101000100000001000100011100010001101110111011011101 0 36 0.40

EST-AR095A11 000101001111011010100000010000101001000000000000100000001100010100110000100010011011011111 0 32 0.36

EST-AR095H03 000110000100000001001120011000002000000002100000200000000000010000001010001110121000010210 6 19 0.21

EST-UNR6162F03 000001000110011001101000011000001101000110101101110000000101010010011100111110111111111110 0 44 0.49

GDI2 000000000100010000110100001011000001010111000000000010001000011000100000001000110100011111 0 27 0.30

ITGB1 100000011100001110011110011001011010001111100110111000000101111110001000111110111110011111 0 50 0.56

ITGB1(secondmarker) 000000000100000100011110011001001010001111100110111000000101111010001000111110111110011111 0 43 0.48

KCTD3 000110101001200100010101011002000001011000000010000000001000011000100000101100010010000101 2 26 0.29

KIAA0042 000010001100001000000000010101211000100011000100101101000000011020110001101120111012011101 4 33 0.37

KIAA0483 000010001100001000010100012100001000100100000000000000000100011100110101111110111011011101 1 33 0.37

KMO 001110201101111001110110111010011010101000000000000000000001010000100010101110000012011111 2 37 0.41

MAP3K8 000001001111100000101100000001001011100101000100100000000100110000110000101100010011011110 0 33 0.37

MLLT10 000001001100000011010010011000001111010100101110110000010100111000011100111110111110111111 0 45 0.50

MSRB2 000001000100000001011010010000000101000100101110110000010100111010001100110110110110111111 0 38 0.42

ADPRT 001210201101111001110110011001011000101010000010000000020001010010101011101110001010011111 3 40 0.44

PFKFB3 000000000100020001110100001010011001010111000000000010000100010000100000120010011100011001 2 26 0.29

PHYH 000001000110011001100000010000001001000110000101110000000100010010011100111110111111111111 0 39 0.43

PIP5K2A 000001000100000001011010011000001101010100001010110000010100111010001000110110011010011101 0 35 0.39

PRTFDC1 000001000110000001011100011000101101000100101111100000000100111010011100111110111111111111 0 45 0.50

PTPRC 000010001100001000011100012000001000100000000100000000000100010000100001101110111010011102 2 26 0.29

Q8WX16 002110101101101100010100011001000001011000000020000000001001011110100000101110010010001101 2 33 0.37

RGS2 000100101000011010010100011000000000000010000010000000000000010000100000112100010010011100 1 22 0.24

RGS2(secondmarker) 002100101100011010010100011000000000000010000010000000000000010000100000110100010010011100 1 23 0.26

S0038 001100101000001000010100011001001111010001000010100000000000010010100000110100010010011100 0 29 0.32

S0039 000001000110011001100000012000001001100100001000110000001100010000010110111010111111011111 1 36 0.40

SSC10G07 000121201110111100110100011001010101100010000000000000000001010110011110201110010110011110 3 38 0.42

SSC25A02 000101001110001200100100011001000101100011000000200000000101010110011110001010010110011100 2 33 0.37

STAM 002101021110011010111000011000101001100000000100110000000100010010111010101110011011011111 2 40 0.44

SVIL 000001001111111010101100000000111011100101000101110000000100010000110000101100010011011110 0 38 0.42

SW1041 000111001110102001110100011001001101101011000000100000001100011100110100101210010010011110 2 39 0.43

SW1103 000001000100011011100100010000001001200101001000100000001100010000010110110010011111011111 1 34 0.38

SW1405 000111001110112001110100011001001101101011000000100000001100011100100100101210010010011110 2 39 0.43

SW1626 002000000100000001210110010001000011110101000100111010001100011000001000111010011100011011 2 34 0.38

SWR1829 000001000110001001010000010000001101000100101111100000000100110000011100110010111111111111 0 38 0.42

SW1894 000110101100000001120010011000001020101000000010000000000000010000000010101112210010010110 4 25 0.28

SW1991 000101001111110010110100001010001011100001000102010000000100011200010000101110010011011110 2 36 0.40

SW2000 000001000110001001100000010000001001000100001000110000001100010000010110110010111111011111 0 33 0.37

SW2043 000001000110011001121100011000101101000110101101110000000100010010011100111110111111111111 1 46 0.51

SW305 000000001100000100010110011001001010001111100110111010000100011010001000110110011110011111 0 40 0.44

SW443 001110101101101101010111011001101010101101001010000001001000011000100000101110010010011110 0 42 0.47

SW497 000010000101001001010110011000011000101010000010000000000002011010101110011110111010010111 1 35 0.39

SW767 001110101001101101010111011001101010101101001010000001001000011000100000101120010010011110 1 40 0.44

SW830 001110101000011010010100011000000100000110100110000000000000010000100000111102010110011100 1 30 0.33

SW920 000001000110011011111100011000001001100101001000100000001101010000010111111110011111011111 0 43 0.48

SW951 000000000100000100010110011001001010001111100110111000000101111010001000111110111110011111 0 42 0.47

SWC19 000010001200001100100100010100011000000111000100001201000000001000110001101122111011011101 4 32 0.36

SWR136 000110101000001000010100011001000101010000000010100000001002011010100000110100010010001101 1 28 0.31

SWR158 000111000010110001110100011001011121101011000000100000001100011100110101101110010010011120 2 40 0.44

SWR334 000111000110110002110100011001001101101011000000000000001100012100110102101110010010011100 3 36 0.40

SWR67 000001010000000000110100001010000001000111000000000010000100010000100000110000011100011001 0 23 0.26

TAF1A 002110101100011001110010211010001010101000000000000000000000010000000010101110010210011111 3 31 0.34

TGFB2 001110101101111101110111011001101011101101001010000001011100011000100000101110010010011110 0 47 0.52

UCHL5 001100101000011011010100011000000100000010000010000000010000010000100000120100010110011110 1 27 0.30

VIM 001101011110011010011000011000101001100001000000010000000101010010111000101110001011011111 0 39 0.43

WAC 000001001111111011111100011001001011100101000101100000000100112000110100101200010011011110 2 41 0.46

WDR26 000110001101011001110110011011011010101000000000000000000000010000100010101010010010011111 0 34 0.38

WDR40A 000101001110011000110100010001010101100011100000000000000101011110001110001110010010011100 0 36 0.40

ZCCHC6 000111001110011100111101011111001100100010001000000100001000010100011110001110011010011110 0 42 0.47

0.41

* Detailed marker information is present in the Supplementary Table 1.

"** Retention fraquency = (no. of ""1"")/90."
